# The relative frequency of pruritus in postherpetic neuralgia patients presenting to the pain clinic and associative factors

**DOI:** 10.1097/MD.0000000000030208

**Published:** 2022-09-02

**Authors:** Cheolhwan Park, Hyunji John, Jaemoon Lee, Seungwan Hong, Minjung Kim, Sangtae Park, Jae Hun Kim

**Affiliations:** a Department of Anesthesiology and Pain Medicine, Konkuk University School of Medicine, Seoul, Korea.

**Keywords:** herpes zoster, itching, neuralgia, neuropathy, postherpetic, pruritus, trigeminal nerve

## Abstract

Postherpetic neuralgia (PHN) is the most common complication of herpes zoster, whereas postherpetic pruritus (PHP) a rare one. Although PHN has been extensively studied, few studies have investigated PHP. The purpose of this study was to investigate PHP incidence and associated factors in patients with PHN. This was a retrospective study of patients with PHN. A total of 645 patients were included. This study conducted in a single university hospital. Data included age, sex, height, weight, pain score, PHN site, medications, nerve blocks, and pulsed radiofrequency treatment. Data also included PHP onset and duration among those with PHP. We divided patients into 2 groups: the control group (group C), comprising those without PHP, and pruritus group (group P), comprising those with PHP. The correlation of PHP with other factors was analyzed. Of 207 patients, 58 were in group P whereas 149 in group C. The mean onset time and duration of PHP were 96.5 and 278.6 days, respectively. Pain scores were lower in group P than in group C after 3 and 4 months following vesicle formation. Patients with PHN in the trigeminal nerve had a higher incidence of PHP compared to those with PHN in others. Twenty-eight percent of patients with PHN developed PHP. At 3 and 4 months after vesicle formation, patients with PHP had greater pain improvement compared to those without. Patients with PHN in the trigeminal nerve also had a higher incidence of PHP compared to others.

## 1. Introduction

The lifetime prevalence of herpes zoster is 20–30%.^[1]^ It is a viral infection caused by reactivation of the varicella-zoster virus. The primary varicella zoster infection occurs when the patient contracts chicken pox. Following the resolution of chicken pox, the virus remains dormant in the dorsal sensory and cranial nerve ganglia for years to decades. Patients are asymptomatic when the virus is dormant. However, reactivation of the varicella-zoster virus results in localized, painful, pruritic, vesicular rashes, usually manifesting unilaterally along the distribution of sensory nerves.^[2]^ Reactivation increases in older or immunocompromised patients, such as those with human immunodeficiency virus infection, malignant tumors, a history of organ transplantation, chemotherapy, or steroid treatment.^[3]^

Postherpetic pruritus (PHP) is another potential complication of herpes zoster.^[4]^ It is a chronic refractory pruritus state in an area previously affected by herpes zoster.^[5]^ Similar to postherpetic neuralgia (PHN), it can degrade the quality of life of patients and cause injury and disability.^[6,7]^ Although PHN has been extensively studied, few studies have investigated PHP. The purpose of this study was to investigate PHP incidence and associated factors in patients with PHN.

## 2. Methods

The Institutional Review Board of the Konkuk University Hospital (IRB file no. KUMC 2020-11-036) approved the study and waived the requirement of obtaining informed consent owing to the retrospective study design. We collected data of patients who had visited the pain clinic of the Konkuk University Hospital and been diagnosed with PHN from January 2011 to June 2020.

We defined PHN as pain continuation for more than 3 months after onset of skin rashes. To observe from the beginning of PHN, inclusion criteria were the diagnosis of PHN and patient presentation within 90 days since the onset of skin rashes.

We excluded data of patients who had visited past 90 days since the onset of skin rashes because initial data for them might be missing or inaccurate. Exclusion criteria were generalized itching or itching in areas without herpes zoster lesions. Data included age, sex, height, weight, pain score, PHN lesions, follow-up duration, medications, nerve blocks, and pulsed radiofrequency treatment. Data also included PHP onset time and duration.

We defined PHP as pruritus only in dermatomes previously affected by herpes zoster. We routinely checked for pain and pruritus whenever the patients with PHN visited the hospital. We also routinely checked the skin of patients complaining of pruritus and confirmed a lack of other skin problems. Through checking skin lesions, it was also found that the range of pruritus was limited to the PHN dermatome.

We also collected the pain score on a numeric rating scale of 0 to 10 (where 0 means “no pain,” and 10 means “worst pain imaginable”). The numeric rating scale was applied 13 times: at onset and after 1, 2, 3, 4, 5, 6, 7, 8, 9, 10, 11, and 12 months.

The location of the lesion was identified as the area of distribution of the trigeminal nerve or cervical, thoracic, lumbar, or sacral dermatomes. As for medications, we investigated tramadol, pregabalin, gabapentin, amitriptyline, nortriptyline, milnacipran, duloxetine, lidocaine patch, and opioid prescriptions. Nerve blocks included the supraorbital nerve, maxillary nerve, mandibular nerve, interscalene brachial plexus, satellite ganglion, cervical epidural, intercostal nerve, thoracic epidural, thoracic sympathetic ganglion, lumbar epidural, lumbar sympathetic ganglion, caudal epidural, and transforaminal epidural blocks. The onset time and duration of PHP were based on the onset of skin rashes.

We divided patients into 2 groups: the control group (group C), comprising those without PHP; and the pruritus group (group P), comprising those with PHP during follow-up. The correlation of PHP with other factors was investigated.

Data were analyzed with SPSS version 17 (SPSS Inc., Chicago, IL). Proportional differences were evaluated using the chi-square test. If there was data with counts <5, Fisher exact test was used instead of chi-square test. The continuous data was assessed for normality using the Kolmogorov-Smirnov test. nonnormally distributed data was analyzed by Wilcoxon rank-sum test. For the cumulative rate of PHP and improvement rate, Kaplan–Meier survival curves were used. A *P*-value < 0.05 was considered to be statistically significant.

## 3. Results

**Figure 1.**
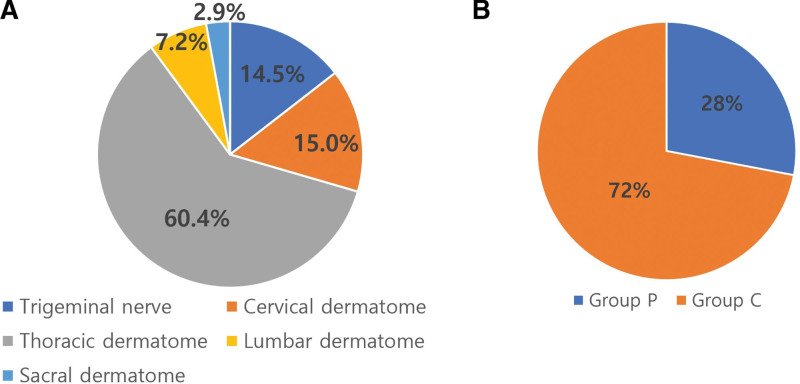
(A) The proportion of lesions related to postherpetic neuralgia. (B) The incidence of postherpetic pruritus. Group C: control group without pruritus; Group P: pruritus group.

In the pain clinic of our hospital, the number of patients who were treated for PHN from January 2011 to June 2020 was 645, and the number of patients who were diagnosed with herpes zoster and PHN through confirmation of skin vesicles was 477. Among them, 207 patients had visited the pain clinic within 90 days since the onset of skin rashes and they were included in this study. The mean follow-up period was 404.4 ± 613.7 days. The lesion locations were the areas of distribution of the trigeminal nerve, cervical, thoracic, lumbar, and sacral dermatomes in 30 (14.5%), 31 (15%), 125 (60.4%), 15 (7.2%), and 6 (2.9%) patients, respectively (Fig. 1A). Groups P and C included 58 (28%) and 149 patients, respectively (Fig. 1B). The data (age, height, weight, pain score, and follow-up duration) did not follow a normal distribution by using the Kolmogorov-Smirnov test (*P* < .05).

**Figure 2.**
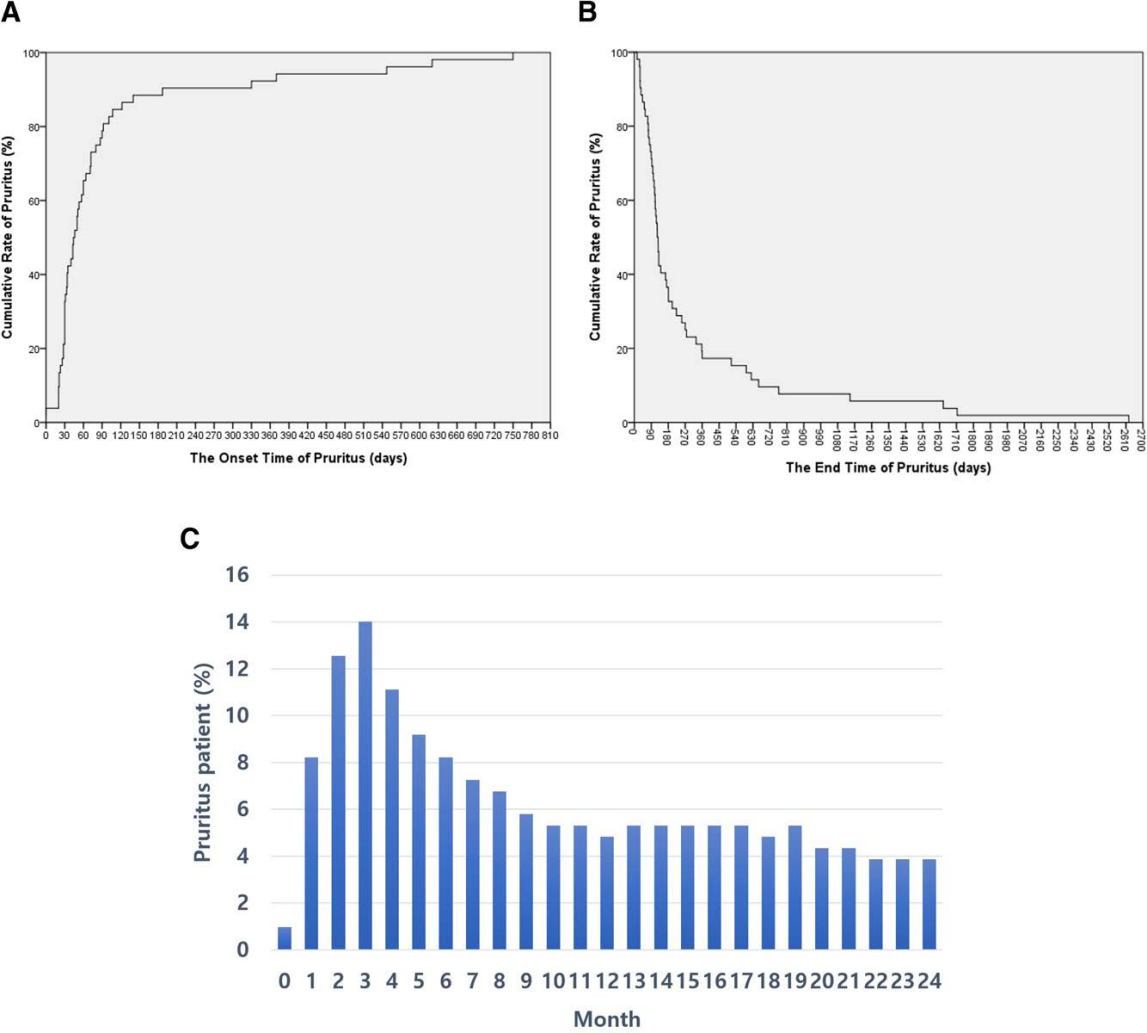
(A). Cumulative rate of postherpetic pruritus in pruritus group. (B) Cumulative rate of complete improvement of postherpetic pruritus in pruritus group. (C) The proportion of patients with pruritus at each period.

The 2 groups showed no significant difference in sex, age, height, weight, follow-up duration, medications, nerve blocks, or pulsed radiofrequency treatment (Table 1). In group P, the mean onset of PHP was 96.5 ± 151.2 days after the onset of skin rashes. Fig. 2A shows the cumulative onset of PHP. In pruritus group, PHP occurred in 79.2% of patients within 90 days and 84.9% of patients within 120 days after the onset of skin rashes (Fig. 2A). Fig. 2B shows the cumulative improvement in PHP. The mean duration of PHP was 278.6 ± 475.3 days. In pruritus group, PHP disappeared from 61.1% of patients within 180 days and 72.2% of patients within 270 days since the onset of skin rashes (Fig. 2B). Fig 2C shows the proportion of patients with pruritus at each period. At the time of 3 months after onset of skin rashes, the proportion was the highest.

**Table 1:** Comparison of sex, mean age, height and weight, medications, nerve blocks, and pulsed radiofrequency treatment between pruritis and control groups.

	Group P (n = 58)	Group C (n = 149)	*P* value
Sex (male/female)	24/34	66/83	0.704
Mean age (yr)	70.8 ± 14.2	71.8 ± 14.1	0.634
Mean height (cm)	159.1 ± 9.0	158.1 ± 12.1	0.599
Mean weight (kg)	57.2 ± 11.1	60.3 ± 13.6	0.160
Mean follow-up duration (d)	467.5 ± 670.4	379.7 ± 590.7	0.361
Medications			
Tramadol	39	100	0.986
Pregabalin	21	63	0.424
Gabapentin	28	56	0.151
Amitriptyline	7	12	0.369
Nortriptyline	22	47	0.381
Milnacipran	1	1	0.487
Duloxetine	1	4	0.686
Lidocaine patch	3	4	0.374
Opioids	5	18	0.477
Nerve block	56	138	0.295
Pulsed radiofrequency	16	37	0.683

Group C: control group without pruritus; Group P: pruritus group.

**Figure 3.**
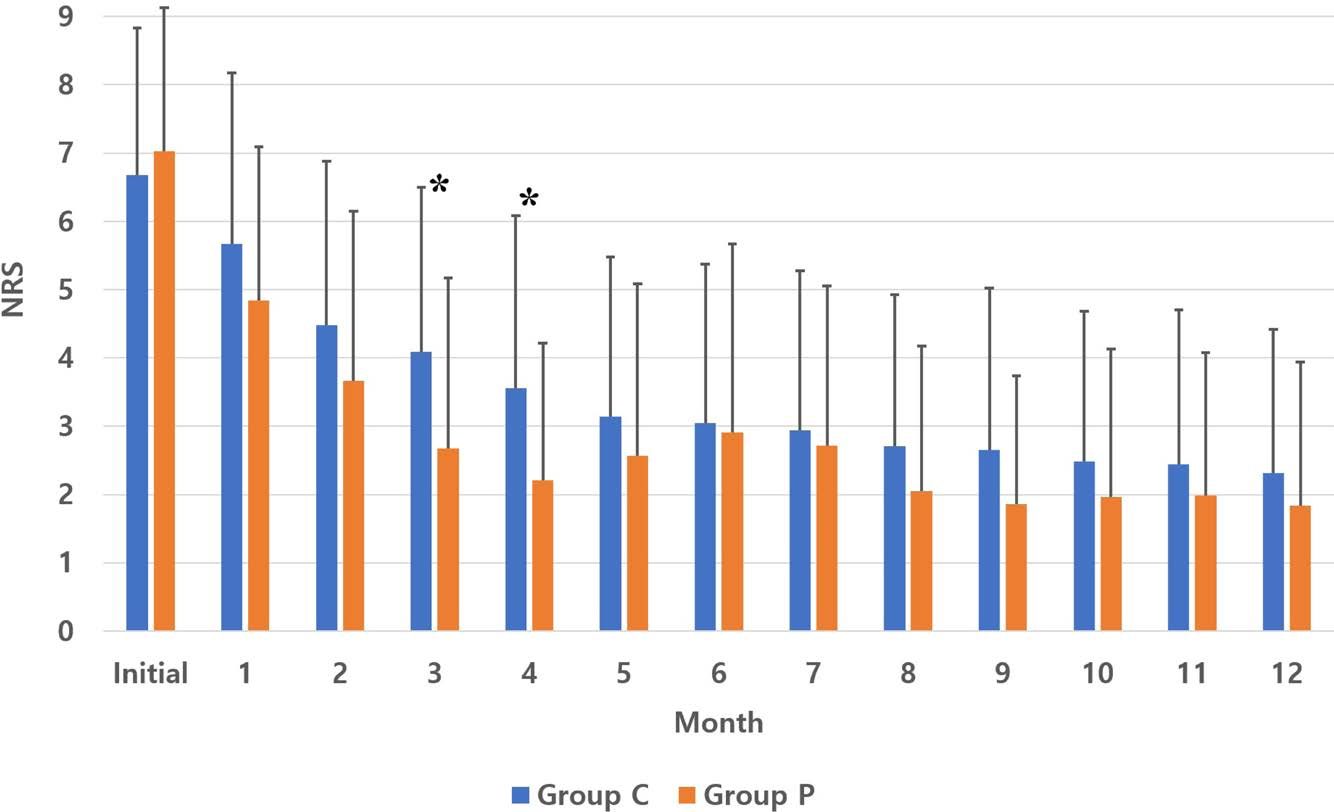
Comparison of pain scores between pruritus and control groups. *The mean pain scores in group P after 3 and 4 months were lower than that in group C (*P* = .005 and 0.030, respectively). Group C: control group without pruritus; Group P: pruritus group.

The 2 groups showed no significant difference in the pain score at onset or after 1, 2, 5, 6, 7, 8, 9, 10, 11, or 12 months. The mean pain scores after 3 and 4 months were 4.08 ± 2.42 and 3.56 ± 2.53 in group C, respectively, and 2.67 ± 2.51 and 2.21 ± 2.00 in group P, respectively, showing significant differences (*P* = .005 and 0.030, respectively; Fig. 3).

**Figure 4.**
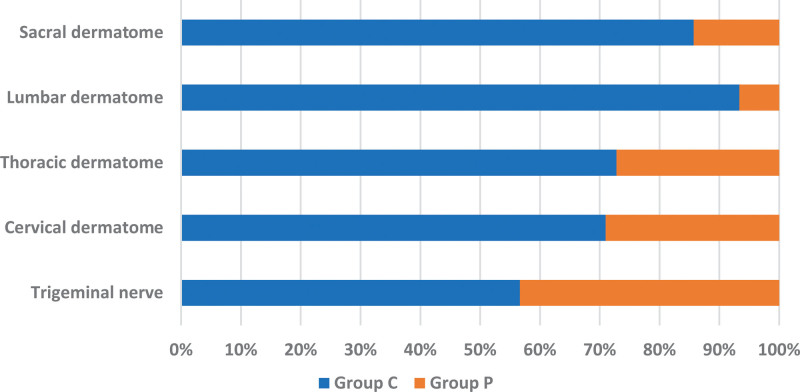
Comparison of pruritus incidence by site. Patients with PHN in the trigeminal nerve had a greater incidence of PHP compared to those with PHN in others (*P* = .043). Group C: control group without pruritus; Group P: pruritus group.

Patients with PHN in the trigeminal nerve had a greater incidence of PHP compared to those with PHN in others. Of 30 patients with herpes zoster in the trigeminal nerve, 13 developed PHP. Of 177 patients with herpes zoster in others, 45 developed PHP (*P* = .043; Fig. 4). Overall, 9 of 31 had PHP in the cervical dermatome, 34 of 125 in the thoracic dermatome, 1 of 15 in the lumbar dermatome, and 1 of 6 in the sacral dermatome (Fig. 4).

## 4. Discussion

In this study, despite no significant difference in the initial pain score, pain scores after 3 and 4 months were lower in pruritus group than in control group. It means that PHP occurred in patients who had highly improved pain after 3 and 4 months. The PHP site is associated with the urge to scratch. PHP is inhibited by pain in relation to the antipruritic effect of scratching.^[8,9]^ Moreover, the inhibition of pain processing can generate PHP,^[8]^ explaining the relationship with better improved pain scores after 3 and 4 months in pruritus group. As shown in Fig. 3, the pain scores at the 3^rd^ and 4^th^ months were significantly different, and the pain scores of the patients with PHP tended to be lower than those of the control group. Although we did not investigate PHP severity, Shimada et al reported a case report of painless severe PHP.^[6]^ Lee et al reported that the severities of pruritus and pain were significantly correlated in a small number of patients (N = 38).^[10]^ On the other hand, Ishikawa et al reported no correlation of the visual analog scale score with PHP. However, they reported that painDETECT questionnaire scores and pruritus were positively correlated in the acute stage (≤30 days) in small number of patients (N = 76).^[11]^

In this study, patients with PHN in the trigeminal nerve had a greater incidence of PHP compared to those with PHN in others. As for the lesion location, the most commonly affected sites for herpes zoster are the thoracic dermatomes.^[12]^ However, in this study, the incidence of PHP was the highest in the trigeminal nerve. Although Lee et al reported no correlation of the lesion location with the incidence of PHP,^[10]^ other studies showed that patients with craniocervical herpes zoster had a greater incidence of PHP.^[11,13]^ The trigeminal nerve and its branches may be vulnerable to PHP. Despite reviewing several documents, the mechanism of trigeminal nerve vulnerability was not identified.

In these results, comparing the 2 groups revealed no association of the occurrence of PHP with age, sex, height, weight, medications, nerve blocks, or pulsed radiofrequency. Oaklander et al reported that age or sex did not affect the occurrence of PHP.^[13]^ Other 2 investigations also showed that age did not affect the occurrence of PHP.^[11,14]^ There is no correlation between PHP and the treatment, which suggests that medications, nerve blocks, or pulsed radiofrequency for herpes zoster or PHN are not the cause of PHP.

PHP is chronic and persistent^[4]^ Although some theories have been proposed, such as spontaneous firing of denervated central nervous system pruritic neurons, imbalance between excitation and inhibition of secondary sensory neurons, and selective preservation of peripheral pruritic fibers from the adjacent unaffected dermatomes, the exact mechanism underlying PHP is unknown.^[15–17]^ It is associated with sensory neuron damage, unlike inflammatory pruritus. Punch skin biopsies from patients with PHN have revealed loss of cutaneous innervation.^[17,18]^ Local activation of a few epidermal nociceptors can cause pruritus by spatial contrast.^[9]^ In peripheral neuropathy, a combination of ongoing or evoked activity from newly regenerating or sparsely surviving nerve branches could generate neuropathic pruritus by the spatial contrast mechanism.^[9]^

The prevalence of PHP varies.^[13]^ In this study, the prevalence of PHP was 28% in patients with PHN who had visited the pain clinic within 90 days since the onset of skin rashes. In different studies, Mittal et al reported that 53 of 100 (53%) patients with PHN experienced PHP, and Ishikawa et al reported that 60 of 76 (78.9%) patients with PHN experienced PHP.^[11,19]^ In our study, the mean follow-up duration was 404.4 days. The mean onset and duration of PHP were 96.5 and 278.6 days after the onset of skin rashes. Studies are limited on the onset and duration of PHP. Asit Mittal et al reported that the onset of PHP varied from immediately following skin rash resolution to several weeks after the resolution of herpes zoster.^[19]^

PHP is less investigated than PHN. The curative treatment for PHP is unknown.^[20]^ Patients with PHP do not respond well to antihistamines. Therefore, treatment of patients with PHP is difficult.^[4]^ However, treatment options for neuropathic pruritus are similar to those for neuropathic pain. Gabapentin and pregabalin are effective in patients with PHP or other types of neurogenic pruritus.^[6,21]^ Gabapentin and pregabalin are used to treat neuropathic pain because they act on signal routes that deal with painful stimuli. They also act on signal routes that deal with pruritic stimuli.^[21]^ There are some reports of the treatment effect for PHP such as pulsed radiofrequency, the stellate ganglion block, acyclovir, topical 2% amitriptyline/0.5% ketamine gel, and carbamazepine with hydroxyzine.^[4,20,22–24]^ Although the treatment of PHP is similar to that of PHN,^[25]^ opioid analgesics should be excluded because they may cause severe PHP in some cases^[22]^ and induce PHP at the spinal level.^[9]^

This investigation has some limitations. First, this study is retrospective study. We checked for pain and pruritus whenever the patients who suffer from postherpetic neuralgia visiting the hospital and put it on the medical records. However, there may be possibility of missing data. Second, we did not investigate the relationship between pain score and severity of pruritus. If trigeminal nerve is vulnerable for PHP, the severity may be higher than other lesions. Further prospective studies enrolling large numbers of patients are needed in the future. Third, there may be possible difficulties to differentiate between pain and pruritus. However, most patients could distinguish pain from pruritus.

In this study, 28% of patients with PHN developed PHP. The pain scores after 3 and 4 months were lower in group P than in group C. Patients with PHN in the trigeminal nerve had a greater incidence of PHP compared to those with PHN in others.

The author declares that there is no conflict of interest.

Funding: This work was supported by National Research Foundation of Korea (NRF) grant funded by the Korea government (MSIT) (No. 2022R1C1C11009176). The funding body had no influence on study design, data collection and analysis, decision to publish, or preparation of the manuscript.

## Author contributions

Conceptualization: Jae Hun Kim

Data curation: Cheolhwan Park, Hyunji John, Jaemoon Lee, Seungwan Hong

Formal analysis: Minjung Kim, Jae Hun Kim

Investigation: Cheolhwan Park, Hyunji John, Jaemoon Lee, Seungwan Hong, Minjung Kim, Sangtae Park, Jae Hun Kim

Supervision: Jae Hun Kim

Validation: Minjung Kim, Sangtae Park

Writing – original draft: Cheolhwan Park, Hyunji John, Jaemoon Lee, Seungwan Hong, Minjung Kim, Sangtae Park, Jae Hun Kim

Writing – review & editing: Jae Hun Kim
